# Gas Permeability through Polyimides: Unraveling the Influence of Free Volume, Intersegmental Distance and Glass Transition Temperature

**DOI:** 10.3390/polym16010013

**Published:** 2023-12-19

**Authors:** Alba Torres, Cenit Soto, Javier Carmona, Bibiana Comesaña-Gandara, Mónica de la Viuda, Laura Palacio, Pedro Prádanos, María Teresa Simorte, Inmaculada Sanz, Raúl Muñoz, Alberto Tena, Antonio Hernández

**Affiliations:** 1 Surface and Porous Materials (SMAP), Associated Research Unit to CSIC, Facultad de Ciencias, Universidad de Valladolid, Paseo Belén 7, E-47011 Valladolid, Spain; alba.torres@uva.es (A.T.); marveliacenit.soto@uva.es (C.S.); fcojavier.carmona@uva.es (J.C.); monicarosa.viuda@uva.es (M.d.l.V.); laura.palacio@uva.es (L.P.); pradanos@termo.uva.es (P.P.); a.tena@uva.es (A.T.); 2 Institute of Sustainable Processes (ISP), Dr. Mergelina s/n, E-47011 Valladolid, Spain; raul.munoz.torre@uva.es; 3 Department of Physics and Inorganic Chemistry, University of Valladolid, Paseo Belén 7, E-47011 Valladolid, Spain; bibiana.comesana@uva.es; 4 UI Cinquima, University of Valladolid, Paseo Belén 7, E-47011 Valladolid, Spain; 5 Department of Organic Chemistry, University of Valladolid, Paseo Belén 7, E-47011 Valladolid, Spain; 6 FCC Medio Ambiente, Avenida Camino de Santiago 40, Edificio 2–Planta 2, E-28050 Madrid, Spain; mariateresasimorte@fcc.es (M.T.S.); inmaculadasv@gmail.com (I.S.)

**Keywords:** membranes for gas separation, d-spacing, free volume, kinetic diameter, glass transition temperature

## Abstract

The relationships between gas permeability and free volume fraction, intersegmental distance, and glass transition temperature, are investigated. They are analyzed for He, CO_2_, O_2_, CH_4_, and N_2_ gases and for five similar polyimides with a wide range of permeabilities, from very low to extremely high ones. It has been established here that there is an exponential relationship between permeability and the free volume fraction, and between permeability and the most probable intersegmental distance as measured by WAXS; in both cases, with an exponential coefficient that depends on the kinetic gas diameter as a quadratic polynomial and with a preexponential positive constant. Moreover, it has been proven that the intersegmental distance increases linearly with the free volume fraction. Finally, it has been established that the free volume fraction increases with the glass transition temperature for the polymers tested, and that they depend on each other in an approximate linear way.

## 1. Introduction

Polyimides play a crucial role in the realm of gas separation membranes, underlining their paramount importance in various industrial applications. Gas separation membranes are essential in processes such as gas purification, carbon capture, and the production of high-purity gases. Polyimides, owing to their unique combination of mechanical strength, thermal stability, and excellent gas permeability, stand out as a preferred material for crafting these membranes. The inherent versatility of polyimides allows for the design and fabrication of membranes with tailored properties, enabling selective gas permeation based on size, shape, and chemical affinity. This selectivity is paramount in industries wherein the separation of specific gases from complex mixtures is imperative. Whether it is enhancing the efficiency of natural gas processing or mitigating greenhouse gas emissions through carbon capture technologies, polyimide-based gas separation membranes contribute significantly to the advancement of environmentally friendly and economically viable processes. The continual research and development in this field underscore the ongoing efforts to optimize polyimide materials for enhanced gas separation performance, further solidifying their indispensable role in shaping the future of sustainable industrial practices.

In general, d-spacing, obtained from wide-angle X-ray diffraction, is accepted to represent intersegmental distance between polymer chains. Long-chain polymers are presumed to have a higher d-spacing value because it seems clear that longer chains induce lower crystallinity leading, for long enough d-spacings, to the transformation from a glassy to a rubbery structure [1]. Moreover, Stadler et al. showed that d-spacing increases with the molecular weight of the polymers [2]. It has also been noted that there is an improvement in gas permeability for longer chain polymers [3]. Therefore, it should be expected that permeability should increase for longer intersegmental distances between polymer chains.

A similar general increase in permeability for increasing free volume fractions seems to be reasonable. Sandhya et al. confirm that, when the gases diffuse through polymeric membranes with low free volume fractions, they cannot penetrate efficiently into the system, thereby decreasing the permeability of the gas [4].

Nevertheless, Bas et al. did not find any clear correlation between neither d-spacing nor free volume with permeability [5]. Park and Paul [6] performed measurements on an extensive collection of rather heterogeneous polyimides and did not find any concluding quantitative dependence of permeability on free volume, perhaps because they used polyimides with similar permeabilities.

We have recently proposed and tested a quantitative correlation of free volume fraction with permeability [7,8,9,10] that we want to verify here for a homologous series of rather similar polyimides with a wide range of permeabilities. We will test as well whether that quantitative correlation can be applied to d-spacing too.

Referring to the dependence of free volume fraction on the glass transition temperature, *T_g_*, Van Krevelen and Nijenhuis [11] reported an increase in free volume with increasing *T_g_* for an extensive assortment of polymers with a positive correlation, although with a considerable scattering. Hensema et al. recognized [12] that the glass transition temperature may be an appropriate way to estimate free volume, although confirming wide deviations from any monotonous fitting line. More recently, White and Lipson [13] showed, by a thermodynamic detailed analysis and experimental testing, that free volume at glass transition temperature must increase in an approximately linear way with *T_g_*. This would justify that, for temperatures below *T_g_*, the same or a similarly linear behavior would hold. This will be tested here for a homologous set of akin polymers, polyimides specifically, with a wide range of permeabilities.

## 2. Materials and Methods

**Figure 1:**
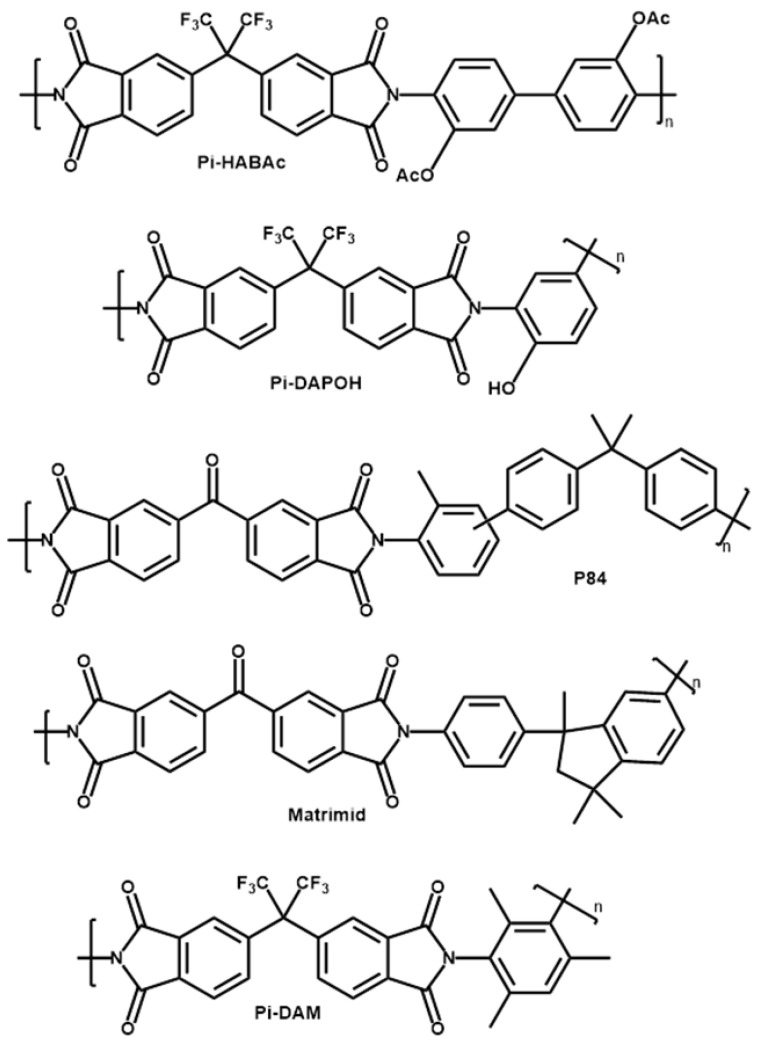
Scheme of the polyimides tested here.

A wide set of commercial (P84^®^ and Matrimid^®^) and other polyimides synthetized by us were used to form films for gas separation. P84^®^ was obtained from HP Polymer GmbH (Lenzing, Austria). Matrimid^®^ was obtained from Huntsman Advanced Materials GmbH (Berkamen, Germany). The synthetized polyimides (Pi-HABAc, Pi-DAM, Pi-DAPOH) were formed by the reaction of 4,4′-(Hexafluoroisopropylidene) diphthalic anhydride, 6FDA, from Fluorochem (Glossop, UK) and 3,3′-Dihydroxybenzidine, HAB, 2,4,6-Trimethyl-1,3-benzenediamine, DAM, both from Apollo Scientific (Manchester, UK) or 2,4-Diaminophenol dihydrochloride, DAP, from Merck-Sigma Aldrich (St. Louis, MO, USA), respectively. Diamines and 6FDA were dried before used. A scheme of the corresponding structures is shown in Figure 1.

Anhydrous 1-methyl-2-pyrrolidinone (NMP), anhydrous pyridine (Py), acetic anhydride, and anhydrous chloroform were purchased from Sigma Aldrich, o-xylene was from VWR (Radnor, PA, USA) and ethanol from Quimilid (Laguna de Duero, Valladolid, Spain). All solvents were used as purchased.

### 2.1. Synthesis of Matrix Polyimides

All the synthetized polyimides were obtained by a two-step polycondensation reaction between 6FDA anhydride and the corresponding diamine reported in previous works [14,15]. A three-necked flask equipped with a mechanical stirrer and gas inlet and outlet was charged with 10 mmol of diamine (HAB, DAM or DAP ·2HCl) and 4 mL of NMP. When using DAP ·2HCl, the salt protection of the amino groups was removed with 100 mmol of pyridine. Then, the mixture was cooled to 0 °C and 10 mmol of 6FDA was added followed by 4 mL of NMP. After stirring for 20 min, the solution was left to warm up to room temperature and left overnight. For HAB and DAM, chemical imidization was carried out by adding 8 mmol of acetic anhydride and 8 mmol of pyridine, left 5 h stirring at room temperature and 1 h at 60 °C to ensure the complete imidization. The resulting polymer was cooled down to room temperature and then precipitated in water, washed firstly with water and afterwards with ethanol and then dried in an oven at 150 °C for 12 h under vacuum. For DAP, an azeotropic imidization was carried out by adding 6 mL of o-xylene to the solution as an azeotropic water remover and it was vigorously stirred and heated for 6 h at 180 °C. During this stage, water was released as a xylene azeotrope. After o-xylene was distilled out from the polymer solution, the solution was cooled down to room temperature and poured on water, washed consecutively with water and ethanol, and then dried at 150 °C for 12 h in a vacuum oven. The synthesized polyimides were designated as Pi-HABAc, Pi-DAM, Pi-DAPOH. The proton NMR spectra were as follows:

Pi-HABAc: ^1^H NMR (400 MHz, DMSO-d_6_) δ 8.20 (d, 2H), 7.98 (d, 2H), 7.84 s, 2H), 7.81–7.75 (m, 4H), 7.66 (d, 2H), 2.14 (s, 6H).

Pi-DAM: ^1^H NMR (400 MHz, DMSO-d_6_) δ 8.21 (d, 2H), 7.99–7.92 (m, 4H), 7.35 (s, 1H), 2.17 (s, 4H), 1.95 (s, 2H).

Pi-DAPOH: ^1^H NMR (400 MHz, DMSO-d_6_) δ 10.33 (s, 1H, OH), 8.20 (dd, 2H), 7.97 (d, 2H), 7.81 (dd, 2H), 7.42 (s, 1H), 7.16 (d, 2H).

The membranes were manufactured by the solution casting method. The solvents used and the drying protocol employed are shown in Table 1. For all the polymers, 10% (*w*/*w*) solutions were prepared in the corresponding solvent. Then, the solution was filtered through a 3.1 µm fiberglass filter (Symta, Madrid, Spain), casted onto a glass plate and slowly heated for solvent evaporation under established conditions.

### 2.2. Gas Separation Transport Properties

A portion of the uniform membrane was loaded into a 25 mm Millipore high-pressure stainless-steel filter holder (Cat. No. XX4502500) (Millipore Corporation, Burlington, MA, USA) as a permeator cell and left one day in vacuum before the measurement, to remove humidity and adsorbed gases in a handmade constant-volume and variable-pressure permeation system. Single gas permeability coefficients (*P_i_*) of He, N_2_, O_2_, CH_4_ and CO_2_ were measured at 35 °C and upstream pressure of 3 bar. Helium permeability was measured at three different pressures (1, 2 and 3 bar) as a protocol to determine the absence of pinholes. The permeability coefficient is typically expressed in barrer [1 barrer = 10^−10^ (cm^3^ (STP) cm)/(cm^2^ s cmHg) = 7.5005 × 10^−18^ m^2^ s^2^ Pa^−1^]. It was obtained with the following equation:

(1) $$P=\frac{273}{76}\left(\frac{LV}{Tp_{a}A}\right)\frac{dp\left(t\right)}{dt}$$

Here, *L* is the thickness of the membranes, *V* is the downstream volume, *T* the temperature, *p_a_* the pressure of the feed gas, *A* the effective area and $\frac{dp\left(t\right)}{dt}$ the slope of downstream versus time. The numeric factors refer to standard pressure and temperature (76 cm Hg y 273.15 K). The thicknesses were measured with a Dualscope MP0R (Fischer Technology, Sindelfingen, Baden Wurtenberg, Germany). The ideal selectivity for a determined gas pair of gases was calculated as the ratio of their single gas permeabilities.

**Figure 2:**
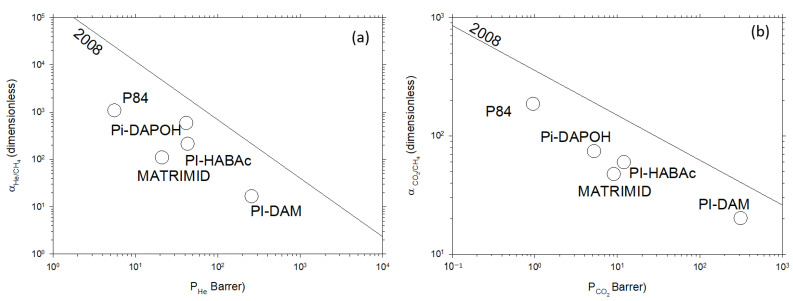
Robeson’s plots for (**a**) He/CH_4_; (**b**) CO_2_/CH_4_ gas pairs for the studied polymers.

Figure 2 shows the Robeson plot exhibiting selectivity, measured as the ratio of permeabilities of the pair of gases to be separated, versus the permeability of the most permeable gas in a double log plot for the pairs He/CH_4_ and CO_2_/CH_4_. The corresponding upper bound straight, as evaluated by Robeson in 2008 [16], is also shown. These kinds of plots are instrumental in assessing the performance of various membrane materials for gas separation applications. Permeability represents the ease with which a specific gas can pass through a membrane, while selectivity reflects the membrane’s ability to distinguish between different gases. The Robeson plots help to create a visualization of the trade-off between these two properties, providing valuable insights into the membrane’s efficiency and guiding the selection of materials for specific gas separation tasks. By plotting the performance of different membranes on a single graph, researchers can rapidly identify the optimal trade-offs and work toward designing membranes with enhanced gas separation capabilities.

The selected membranes, a homologous series of polyimides with rather similar structures, cover a rather wide range of permeability and have selectivities that place their representative points in Robeson’s plots along lines parallel to the successive Robeson limits (selectivities decreasing with permeability increasing), particularly with the 2008 one, as shown in Figure 2.

## 3. Theory

### 3.1. Free Volume Fraction

**Figure 3:**
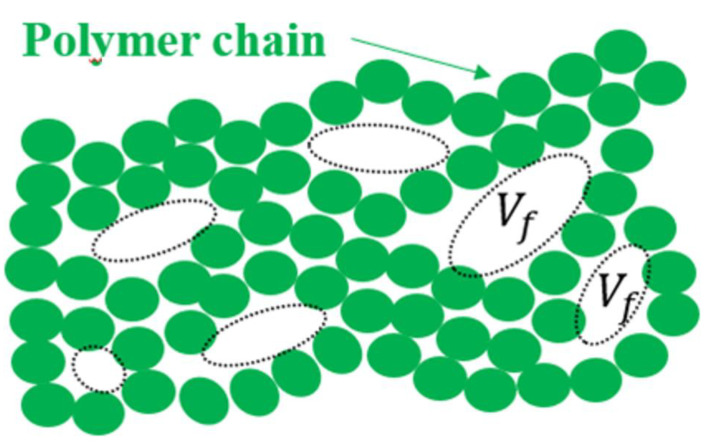
Scheme of free volume or inter-chain holes contributing to the total free volume.

Free volume in a polymer is the portion of the total volume that is not occupied by the polymer chains themselves, allowing for the movement of diffusing molecules. It is typically understood to refer to the spaces or pores between polymer chain segments. The concept is schematically shown in Figure 3. Free volume may depend on the size of the gas molecules permeating the membrane, because free volume should refer to the volume occupiable by the gas molecule to be transported. In our case, all the gases have very similar sizes and all of them should detect similarly small voids opened for transport in such a way that their sizes will not be taken into account to evaluate the free volume.

Densities of the materials can be determined by using a CP225 Analytical Balance from Sartorius (Sartorius, Göttingen, Germany) equipped with a density measurement kit using the Archimedes principle. The Archimedes principle states that a body immersed in a fluid experiments a buoyancy force acting upwards that equals the weight of the fluid displaced by the body. Therefore, the average density can then be obtained as

(2) $$\rho =\rho_{C_{8}H_{18}}\frac{W_{air}}{W_{air}-W_{C_{8}H_{18}}}$$

Here, $\rho_{C_{8}H_{18}}$ corresponds to the isooctane’s density, $W_{air}$ is the sample weight in air and $W_{C_{8}H_{18}}$ stands for the weight of the sample when submerged in isooctane. This method corresponds to the standard ISO 1183-1/ASTM D792 that requires weighing the samples at room temperature, both in air and convenient liquid. Here, isooctane was chosen as the immersion liquid because it is not absorbed by most polymers, it is not hygroscopic and it does not tend to form bubbles.

The most common method used to evaluate the free volume fraction, FFV, which will be referred to as $f\left(f\equiv FFV\right)$ hereafter for easy notation, can be described as follows:

(3) $$f=\frac{V-V_{0}}{V}$$

Here, V=1/ρ (ρ being the density) and $V_{0}$ is the volume of the chain per unit mass. $V_{0}$ can be obtained from the van der Waals [11,17,18,19] specific volume, $V_{w}$, as

(4) $$V_{0}=1.3V_{w}$$

The van der Waals volume can be evaluated by using the Bondi’s group contribution theory [11] or by molecular modeling of the polymer repeating units, with programs like Hyperchem Molecular Modeling (Hypercube, Gainesville, FL, USA) [20,21] or DS BIOVIA Materials Studio software (2023 v23.1.0.3829) (BioVia Dassault Systémes, San Diego, CA, USA) [8,22].

Within the frame of the so-called solution diffusion theory, permeability can be written as P=SD (the product of solubility *S* and diffusivity *D*). Thornton et al. [23] proposed a dependence of on as given by $D=A_{D}e^{\alpha_{D}f}$, Previously, a Doolite type of dependence of *D* on *f* ($D=Ce^{-C^{\prime }/f}$), originally used by Fujita [24] and Lee [25], was accepted. Fractional free volume, *f*, was moved from the denominator in a Doolittle’s type correlation for the diffusion coefficient to the numerator in the exponent to account for the effect of occupied volume on gas diffusion. Thus, permeability can be written as

(5) $$P=SD=Ae^{\alpha f}$$

Here, we distinguish between the exponential and preexponential factors for diffusivity, $A_{D}$ and $\alpha_{D}$, and those for permeability, A and α. It can be assumed that Equation (5) holds when the solubility is almost independent of *f* or depends, like diffusivity, exponentially on *f* [12]. Several models admit a linear dependence of the exponential constant (α in Equation (5)) on the square of the gas kinetic diameter, *d_k_*. These models are based on a reasonable linear dependence of the diffusion activation energy on the transversal area of the penetrant founded, which is reasonable when considering the hard sphere diffusion model wherein diffusion depends on the cross-section area of diffusing molecules [26]. Nevertheless, for the sake of comprehensiveness, a polynomial will be tested here including a linear summand [27]. Thus, using a quadratic polynomial for the constant in the exponent of Equation (5):

(6) $$\alpha =a+bd_{k}+cd_{k}^{2}$$

The kinetic diameters can be taken from Breck [28], which are widely used. It is true that, as pointed out by Matteucci et al. [29], the value reported as the kinetic diameter by Breck, for example, for CO_2_, 3.3 Å, is significantly lower than the Lennard–Jones collision diameter (4.05 Å), but Breck himself rationalized this low value on the basis of experimental data of CO_2_ adsorption on zeolites with known sizes. The shortcomings of Breck’s data have, in fact, led to several alternative proposals of effective molecular sizes leading to different suitable space scales [25,30,31,32]. Nevertheless, here we will use Breck’s kinetic diameters attending to their common usage.

Combining Equations (5) and (6), we obtain

(7) $$lnP=lnA+\alpha f=\left[lnA+af\right]+\left[bf\right]d_{k}+\left[cf\right]d_{k}^{2}$$

If constants in Equation (6) are assumed as equal for a given ensemble of polymers, the free volume can be referenced to a given polymer by correlating bf with those of this polymer, and if its free volume fraction is known, we could evaluate all f for the polymers in the ensemble [27].

### 3.2. Glass Transition Temperature

The glass transition temperature, *T_g_*, was determined by using a Differential Scanning Calorimeter DSC 25 from TA Instruments (Waters Co., New Castle, DE, USA). Samples were prepared by encapsulating a single membrane disc using Tzero^®^ Aluminum crucibles from TA Instruments with a nominal mass of 52 mg. Each lid and pan mass were weighted separately with a resolution of ±0.001 mg and were selected to obtain a mass difference between the reference and the empty crucible always lower than ±0.02 mg. Samples, with masses between 0.6 and 2.0 mg, were determined with an error smaller than 0.005 mg. This procedure complies with the instructions of the ISO 11357-2:2020(E) standard. As the glass transition temperatures are high, a preliminary thermal cycle is performed until reaching a temperature high enough to erase the previous thermal history of the material, and afterwards *T_g_* is evaluated in a second heating cycle. The reported *T_g_* are *T*_*i*,*g*_ (corresponding to the inflection point). All heating and cooling cycles were carried out at a rate of 20 K min^−1^.

### 3.3. Intersegmental Distance between Polymer Chains

**Figure 4:**
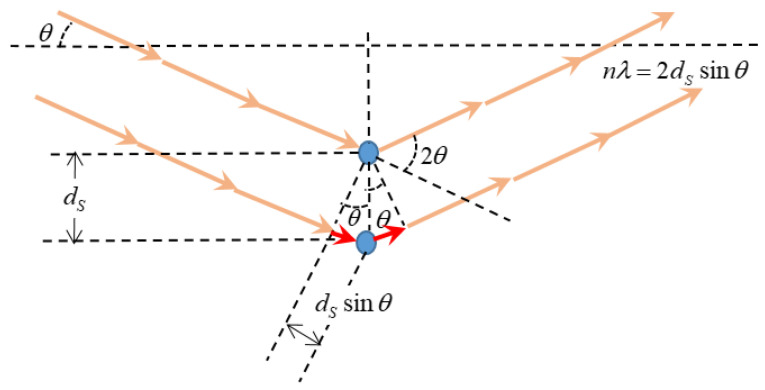
X-ray diffraction and Bragg’s law.

Wide-angle X-ray scattering (WAXS) was recorded at room temperature by means of a Bruker D8 discover A25 advanced diffractometer equipped with a Goebel mirror with of Cu Kα (λ = 1.542 Å) as the radiation source (Bruker, Billerica, MA, USA). The system worked with a LynxEye detector using a step-scanning mode ranging from 5° to 70° (with time periods of 0.5 s and a 2θ pace of 0.020°). The preferential segmental distance ($d_{S}$) in the chain-packing of the amorphous polymers was determined using Bragg’s Law according to Equation (8) which refers to Figure 4:

(8) $$n\lambda =2d_{s}sin\theta$$

Here, θ is the scattering angle and, as can be inferred from Figure 4 and Equation (8), smaller angles correspond to longer segmental distances. A plot of the X-ray intensity versus the scattering angle give a certain distribution that, according to Equation (8), for a given wavelength, could be transformed into a $d_{S}$ distribution. Due to the fact that the polymers tested here are not crystalline, there are wide statistically distributed intersegmental distances showing a most probable $d_{S}$ that can be taken as representative of the whole distribution of intersegmental distances.

As mentioned, it seems reasonable that an increase in the mean segmental distance would lead to an increase in the gas permeability [33,34]. For example, it is known that the presence of bulky pendant groups in the backbone chain of polymers typically increases $d_{S}$ with a simultaneous rising of rigidity and permeability [34], although neither the correlation nor any form of analytical correlation has been universally recognized so far. Even some researchers report that $d_{S}$ does not always correspond to the intermolecular distance governing the diffusivity or permeability of the gas [35].

This work will assume a tentative exponential dependence of permeability on $d_{S}$ according to

(9) $$P=Be^{\beta d_{S}}$$

giving a similar dependence to that shown in Equation (5) for P on *f*. Moreover, by analogy with Equation (6), we can assume a quadratic dependence of *β* on $d_{k}$:

(10) $$\beta =a^{\prime }+b^{\prime }d_{k}+c^{\prime }d_{k}^{2}$$

Leading to

(11) $$lnP=\left[lnB+a^{\prime }d_{S}\right]+\left[b^{\prime }d_{S}\right]d_{k}+\left[c^{\prime }d_{S}\right]d_{k}^{2}$$

Analogously to what was deduced from Equation (7), we can see that a plot of permeability as a function of $d_{k}$ would allow for the evaluation of $d_{S}$ by assuming now that the parameters in Equation (10) are invariable.

On the other hand,

(12) $$\left[lnA+af\right]+\left[bf\right]d_{k}+\left[cf\right]d_{k}^{2}=\left[lnB+a^{\prime }d_{S}\right]+\left[b^{\prime }d_{S}\right]d_{k}+\left[c^{\prime }d_{S}\right]d_{k}^{2}$$

This relationship states that FFV would be linear with $d_{S}$:

(13) $$f=\frac{lnB-lnA}{a+bd_{k}+cd_{k}^{2}}+\frac{a^{\prime }+b^{\prime }d_{k}+c^{\prime }d_{k}^{2}}{a+bd_{k}+cd_{k}^{2}}d_{s}$$

Therefore, the free volume fraction should be linear with d-spacing:

(14) $$f=\Phi \left(d_{k}\right)+\Psi \left(d_{k}\right)d_{s}$$

With intercept and slope depending on the gas through its kinetic diameter according to Equations (15) and (16), respectively,

(15) $$\Phi \left(d_{k}\right)=\frac{lnB-lnA}{a+bd_{k}+cd_{k}^{2}}$$

(16) $$\Psi \left(d_{k}\right)=\frac{a^{\prime }+b^{\prime }d_{k}+c^{\prime }d_{k}^{2}}{a+bd_{k}+cd_{k}^{2}}$$

Note that the units for the used parameters are

(17) $$\begin{array}{c} a\to dimensionless\;a^{\prime }=1/Å\\ b\to 1/Å\;b^{\prime }=1/{Å}^{2}\\ c\to 1/{Å}^{2}\;c^{\prime }=1/{Å}^{3} \end{array}$$

Consequently,

(18) $$\Phi \left(d_{k}\right)\to dimensionless\;\Psi \left(d_{k}\right)\to 1/Å$$

where it is assumed that f is the fraction (from 0 to 1) of free volume, and $d_{S}$ and $d_{k}$ in Å.

## 4. Results

### 4.1. Free Volume

**Figure 5:**
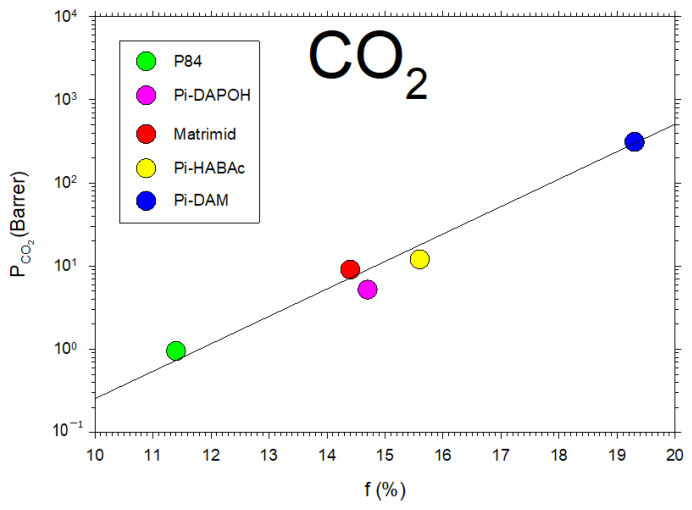
CO_2_ permeability versus the free volume for the membranes studied here.

In Figure 5, we show the permeability of CO_2_, for the membranes that we studied here, as a function of f showing the fitted straight line, corresponding to Equation (5). These results for f, as obtained by using the Biovia Materials Studio software(DS BIOVIA Materials Studio 2023 v23.1.0.3829), approximately agree with the values collected from the literature when possible [36,37,38]. Molecular dynamics simulations make it possible to estimate FFV by assuming a big enough number of polymer chains that are left to relax inside a box of a given size and using a probe molecule to determine the free volume [39]. This approach allows for accounting for the potential interchain interactions on the packing structure. Additionally, some studies gave comparable values by molecular simulation and via Bondi’s method for free volume fractions [39]. Given that Bondi’s group contributions are not kept truly updated [6,11,40], it seems preferable to use molecular simulations to obtain more accurate estimations of FFV.

**Figure 6:**
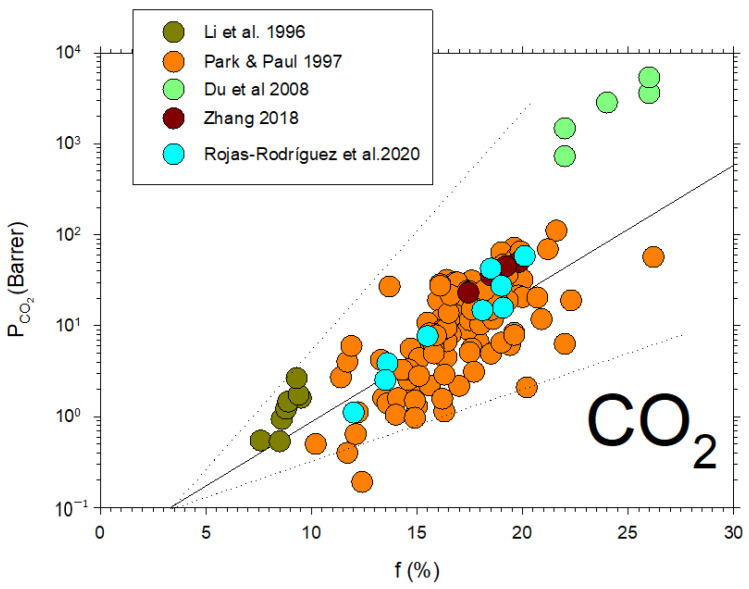
CO_2_ permeability versus the free volume fraction from previous literature [6,41,42,43,44].

CO_2_ permeability as a function of the fraction of free volume for several data extracted from the literature [6,41,42,43,44] is shown in Figure 6.

**Figure 7:**
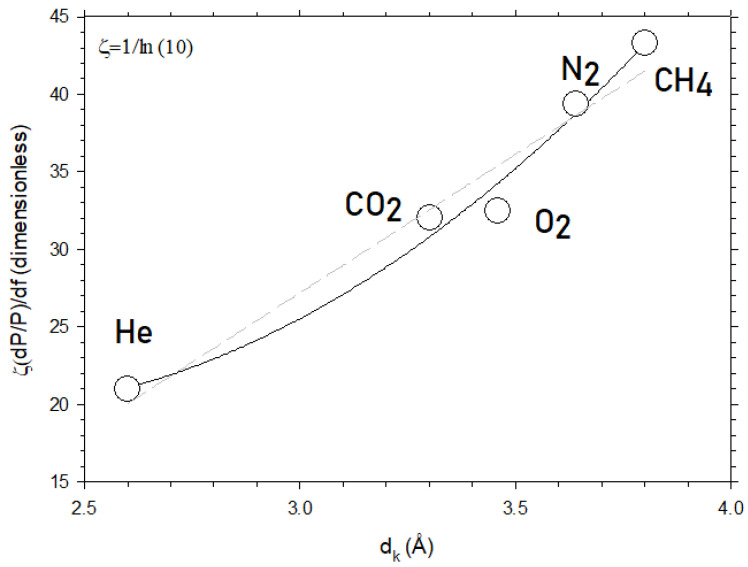
The slope of ln P for the membranes studied here versus f as a function of d_k_, i.e., for different kinetic diameters of the permeant. The dashed straight line corresponds to the linear fitting while the continuous line is the best parabolic fitting.

Figure 7 shows the slope of Figure 5 for CO_2_ and for the other gases studied here as a function of their kinetic diameter. Note that, in Figure 7, the ordinates correspond to the slope of versus which is proportional to $\alpha =a+bd_{k}+cd_{k}^{2}$, in accordance with Equations (5) and (7). The constant appears in order to pass from logP to lnP because $\log P=\frac{\ln P}{\ln10}=\zeta \ln P\;$and the slope of logP versus f is $d\log P/df=\zeta d\ln P/df=\zeta \frac{dP/P}{df}=\zeta \alpha$.

According to Equation (7), this slope should be quadratic with d_k_, as effectively shown in Figure 7. This confirms that Equation (7) captures the essence of the dependence of permeability on the free volume fraction. Note that the linear dependency could also be possible but with a lower fitting goodness of 0.938 as compared to 0.983 for the quadratic dependence. The values of the parameters of Equation (7) obtained by fitting are shown in Table 2.

### 4.2. Free Volume Fraction and Glass Transition Temperature

**Figure 8:**
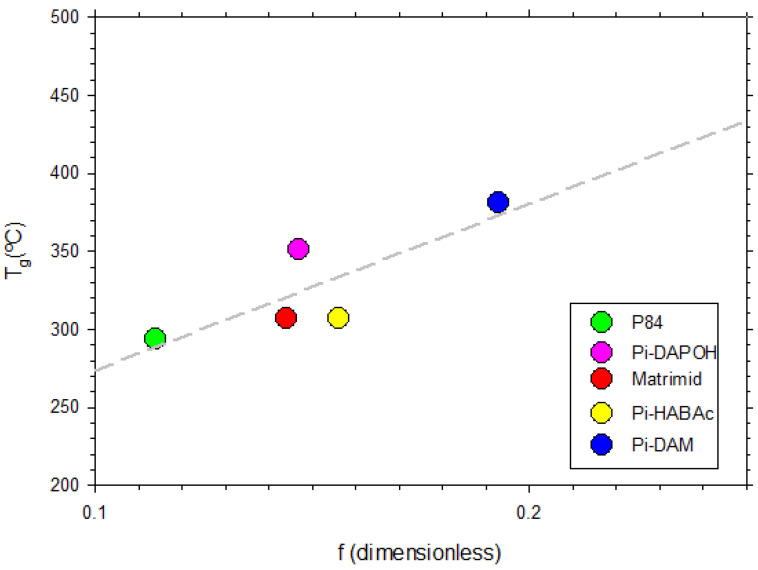
The glass transition temperature as a function of the free volume fraction. The straight line can only be taken as an eye guide.

To test the dependence of the glass transition temperature on the fraction of free volume, we plot both of these magnitudes in Figure 8, where it seems clear that they are positively correlated. This dependency is in fact only roughly linear (correlation index < 0.8). This can only be considered as a hint of a tendency of glass transition temperature to increase with free volume or vice versa.

It is crucial to keep in mind that we are working with extremely rigid (high Tg) polymers, meaning that chain segments are only given very little mobility. Free volume thus appears as spaces between rigid chains, and more rigid polymers—and occasionally branched ones—are beneficial because they provide larger free volumes and wider pathways for gases to permeate.

### 4.3. Intersegmental Distance between Polymer Chains

**Figure 9:**
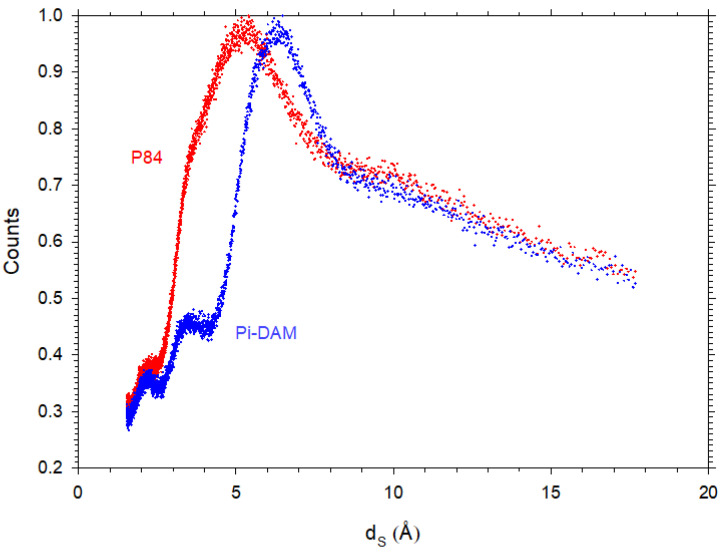
d-Spacing distribution for P84 and Pi-DAM showing the amorphous nature of our polymers. Note that the counts have been normalized to 1 for the most probable d-spacing.

In Figure 9, two examples of d-spacing obtained by WAXS are presented. It is worth noting that they are statistically distributed in accordance with the amorphous nature of polyimides.

**Figure 10:**
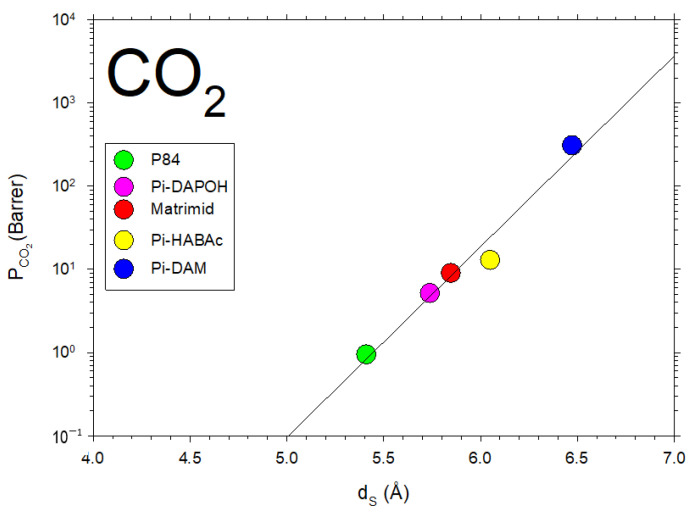
CO_2_ permeability as a function of d_S_ for the membranes studied in this article.

In Figure 10, the permeability of CO_2_, for the membranes we studied here, as a function of $d_{S}$ showing the fitted straight line corresponding to Equation (9), is shown.

**Figure 11:**
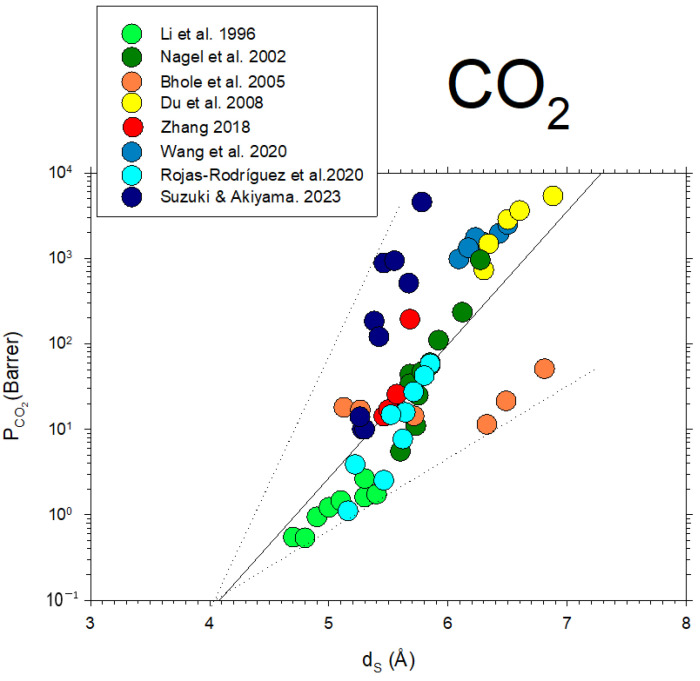
CO_2_ permeability as a function of d_S_ from the literature [42,43,44,45,46,47,48,49].

Some data from the literature [42,43,44,45,46,47,48,49] on CO_2_ permeability versus d-spacing are described in Figure 11.

Note that, although in a somehow diffuse way, a certainly linear dependence of P on $d_{S}$ with some divergences could be due to the possible non-homologous series of membranes shown in Figure 11. Better fittings are clearly shown for the membranes studied here, as shown in Figure 10.

**Figure 12:**
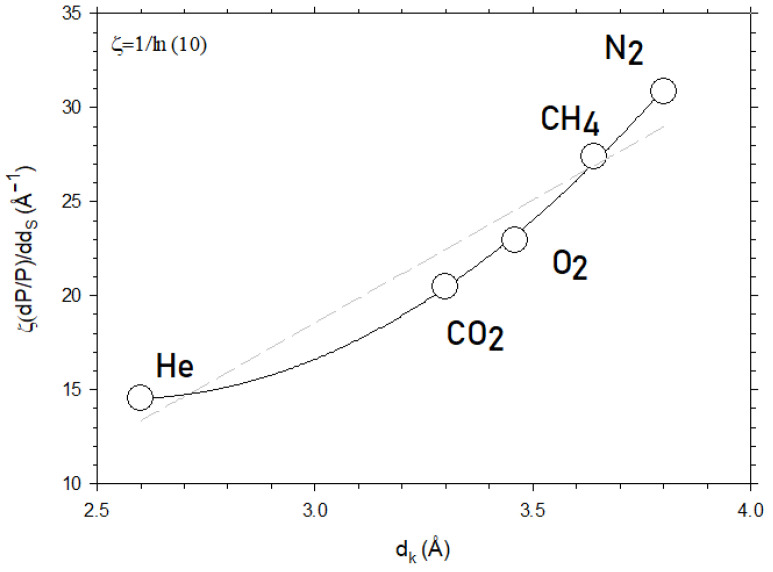
The slope of ln P versus d_S_ as a function of d_k_, i.e., for different kinetic diameters of the permeant. The dashed straight line corresponds to the linear fitting while the continuous line is the best parabolic fitting.

In Figure 12, we plot the slope of Figure 10 for CO_2_ and for the other gases studied here as a function of their kinetic diameter. According to Equation (11), this slope should be quadratic with d_k_, as effectively shown in Figure 12. Note that the linear dependency could also be possible but with a lower fitting goodness of 0.925, as compared to 0.998 for the quadratic dependence.

In Figure 12, the ordinates correspond to the slope of logP versus $d_{S}\;$, which is proportional to $\beta =a^{\prime }+b^{\prime }d_{k}+c^{\prime }d_{k}^{2}$ in accordance with Equations (9) and (11). The constant ζ=1/ln10 appears in order to pass from logP to lnP because $\log P=\frac{\ln P}{\ln10}=\zeta \ln P\;$ and the slope of logP  versus $d_{S}$ is $d\log P/dd_{S}=\zeta d\ln P/dd_{S}=\zeta \frac{dP/P}{dd_{S}}=\zeta \beta$. The values of the parameters of Equation (11) obtained by fitting are shown in Table 3.

### 4.4. Fractional Free Volume and d-Spacing

**Figure 13:**
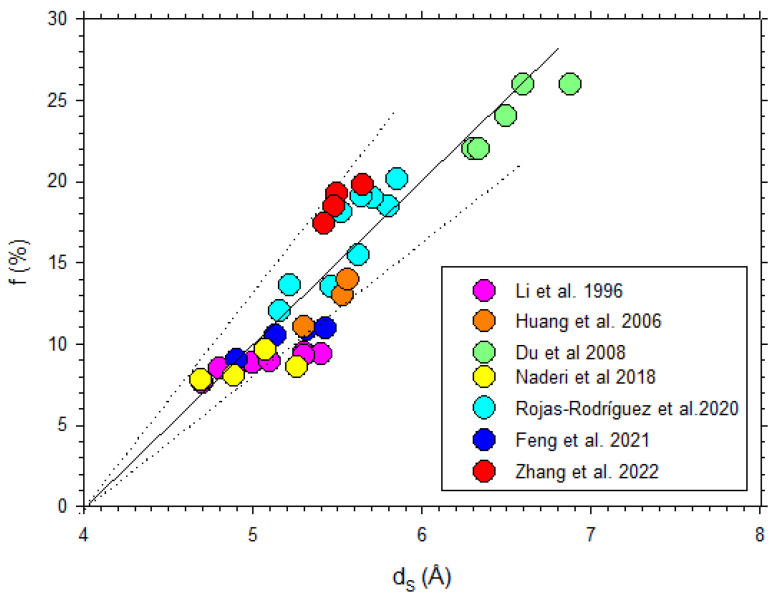
Fractional free volume as a function of $d_{S}$ from previous literature [42,44,45,50,51,52,53].

Some data from the literature [42,44,45,50,51,52,53] on the free volume fraction versus d-spacing are shown in Figure 13, where it is seen that there is an average linear trend according to Equation (14).

**Figure 14:**
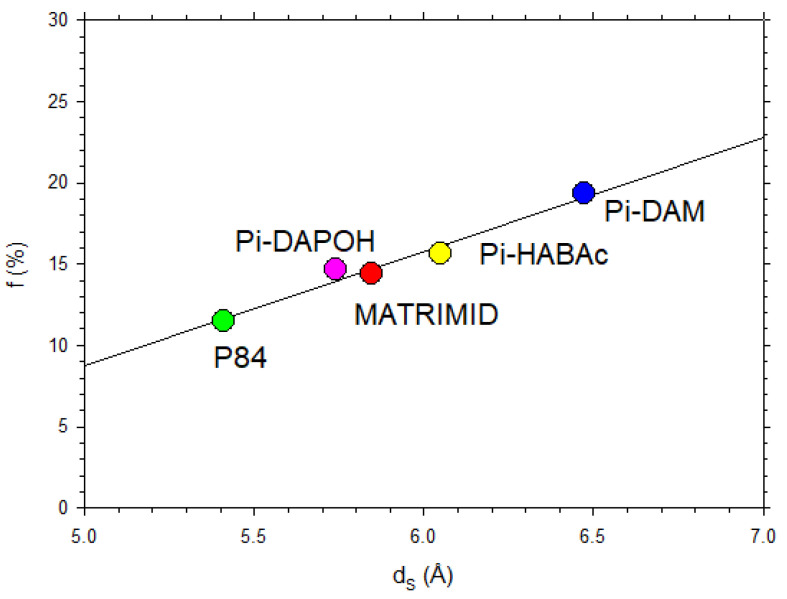
Fractional free volume as a function of $d_{S}$ for the membranes studied in this article.

Figure 14 shows f versus $d_{S}$ for our polyimide membranes showing a clear linear dependence.

Equations (15) and (16) state that f would depend on the permeated gas. In our case, the free volume f has been evaluated without taking into account the fraction of void volume effectively accessible to each gas; therefore, the constants Φ and Ψ would not depend on $d_{k}$. This, attending to Equations (15) and (16), would mean that

(19) $$\left.\begin{array}{c} \frac{a+bd_{k}+cd_{k}^{2}}{lnB-lnA}=C_{1}\\ \frac{a^{\prime }+b^{\prime }d_{k}+c^{\prime }d_{k}^{2}}{a+bd_{k}+cd_{k}^{2}}C_{2} \end{array}\right\}$$

In fact, the fitted straight line in Figure 13 corresponds to

(20) $$f=-0.26+0.0701d_{S}$$

While the corresponding ratios of the parameters of Equations (7) and (11) are, according to Table 1 and Table 2,

(21) $$\left.\begin{array}{c} a^{\prime }/a=0.076\\ b^{\prime }/b=0.074\\ c^{\prime }/c=0.084 \end{array}\right\}$$

The accordance means that, in $f=\Phi \left(d_{k}\right)+\Psi \left(d_{k}\right)d_{S}$, the slope $\Psi \left(d_{k}\right)=\frac{a^{\prime }+b^{\prime }d_{k}+c^{\prime }d_{k}^{2}}{a+bd_{k}+cd_{k}^{2}}$ does not depend on $d_{k}$. The ordinate intercept $\Phi \left(d_{k}\right)=\frac{lnB-lnA}{a+bd_{k}+cd_{k}^{2}}$ does not depend on $d_{k}$ either because A and B depend on $d_{k}$, approximately compensating for the dependence of the denominator. In fact, the extreme values of Φ are −0.24 and −0.30, which averaged give −0.27, and this compares nicely to the ordinate intercept in Equation (20).

## 5. Conclusions

It has been analyzed how free volume fraction, intersegmental distance and glass transition temperature are correlated to each other and with gas permeability for several simple gases including He, CO_2_, O_2_, CH_4_ and N_2_. This was achieved by using a series of similar polyimides covering a wide range of permeabilities from rather low to very high ones.

In effect, it has been proved that the correlation of permeability with free volume fraction and intersegmental distance are both rather similar exponentials, indicating that permeability increases exponentially with both the free volume fraction and the intersegmental distance. It has also been shown that the pre-multiplicative factors in the exponents depend, in both cases, on the kinetic gas diameter as a quadratic polynomial. Positive preexponential elements are present in both relationships.

It is important to point out that no theoretical background has been proposed for these correlations here. Specifically for the quadratic dependence of α on $d_{k}$, while no theory had been proposed in the literature for the dependence of permeability on d-spacing. But the correlations tested here have at least a clear phenomenological value. While a convenient justification could be rather interesting, it was not our objective here.

It is worth noting that, because the polymers tested here are amorphous, there are relatively wide statistical distributions for the intersegmental distances that lead to not sharply defined d-spacing values. Moreover, it is also worth considering that free volume is a somehow ambiguous concept that has here been taken as defined by the voids left between the polymer backbones and evaluated by molecular dynamics and density measurements. These factors can explain the difficulties in detecting the dependencies tested here within the literature on the topic. It is also clear that the long-range dependencies can coexist with short-range ones with rather different behaviors, especially when taking into account data from different sources and for rather different polymers.

It has been also confirmed that free volume fraction and intersegmental distance are both linearly positively dependent on each other. This means that there are specific correlations, that have been tested here, between the constants involved in both exponentials.

The existence of monotonous increasing correlations between permeability and free volume fraction and intersegmental distance seems reasonable and has been made plausible in the literature but was never analyzed in depth. The relevance of such correlations is clear when designing polymers for gas separation and should clarify the structure versus function of gas transport through polymeric membranes. With this objective, the correlations shown here should be tested for the specific class of polymers to be used. Of course, some of the details of these relationships should be specific for the polymers studied, but although the details could differ, in our opinion the general trends must be true for any analogous polymeric series, probably excluding polymers with strong affinities for the penetrant.

To conclude, it has been shown that the glass temperature increases with the free volume fraction in our case. Of course, this complies with the largely admitted idea of obtaining higher permeabilities for more rigid glassy polymers. This correlation was never clearly analyzed and could depend on the class of polymers studied.

## Figures and Tables

**Table 1:** Solvents used during the film formation for the studied polymers.

Polymer	Solvent	Drying
Matrimid^®^	THF	Room temperature until dry and 120 °C for 12 h under vacuum to complete solvent evaporation
P84^®^, Pi-HABAc, Pi-DAPOH and Pi-DAM	NMP	At 60 °C for 12 h and 100 °C for 1 h. Finally, until 300 °C under N_2_ atmosphere ^a^

^a^ Heating protocol: 5 °C/min at 150–200-250–300 °C holding 1 h at each temperature.

**Table 2:** Fitted values for the parameters in Equation (7).

a (Dimensionless)	b (1/Å)	c (1/Å^2^)
146.61	−92.66	21.11

**Table 3:** Fitted values for the parameters in Equation (11).

a′ (1/Å)	b′ (1/Å^2^)	c′ (1/Å^3^)
1939.47	−1255.83	245.68

## Data Availability

All the relevant data for the research here shown are included within the paper.
